# Whole-Genome Sequencing of *Vibrio cholerae* O1 El Tor Strains Isolated in Ukraine (2011) and Russia (2014)

**DOI:** 10.1128/genomeA.01640-16

**Published:** 2017-02-23

**Authors:** Nina I. Smirnova, Yaroslav M. Krasnov, Elena Y. Agafonova, Elena Y. Shchelkanova, Zhanna V. Alkhova, Vladimir V. Kutyrev

**Affiliations:** Federal Government Health Institution Russian Researh Anti-Plaque Institute “Microbe,” Saratov, Russia

## Abstract

Here, we present the draft whole-genome sequence of *Vibrio cholerae* O1 El Tor strains 76 and M3265/80, isolated in Mariupol, Ukraine, and Moscow, Russia. The presence of various mutations detected in virulence-associated mobile elements indicates high genetic similarity of the strains reported here with new highly virulent variants of the cholera agent *V. cholerae*.

## GENOME ANNOUNCEMENT

*Vibrio cholerae* is the etiologic agent of cholera, а severe diarrheal disease. Pandemic *V. cholerae* strains of the O1 serogroup have two biotypes: classical and El Tor. Isolates from the current seventh cholera pandemic are mostly of the El Tor biotype. The principal virulence determinants are cholera toxin and toxin-coregulated pili (1). Recent studies of the genome of* V. cholerae* strains from previous and current epidemics have demonstrated that genomic changes and alteration in the cholera toxin prophage (CTX) of typical El Tor strains resulted in the emergence of new variants (2–4). Here, we report the first draft whole-genome sequences of two* V. cholerae* O1 El Tor strains. The first strain is* V. cholerae* 76, isolated from a patient during an outbreak of cholera in Mariupol, Ukraine, in 2011. The second is the toxigenic *V. cholerae* strain M3265/80, isolated from a patient who arrived in Moscow, Russia, from India in 2014.

The libraries were prepared using Ion PGM preparation protocols (Thermo Fisher Scientific Inc., USA) and then sequenced on the Ion PGM platform, generating 1.0 million (~200-bp) reads (35× genome coverage) and 1.8 million (~400-bp) reads (58× genome coverage) for 76 and M3265/80, respectively. *De novo* genome assembly was performed using Newbler version 2.6. Draft genomes were annotated using the NCBI Prokaryotic Genome Annotation Pipeline.

The draft genome of 76 (4,001,091 bp; 47.5% GC content) consists of 265 contigs, and 3,572 genes and 51 tRNAs were identified. The draft genome of M3265/80 (4,017,694 bp; 47.5% GC content) consists of 123 contigs, with 3,729 genes and 58 tRNAs identified.

A comparative analysis of the virulence-associated mobile elements of strain 76—such as prophages CTXφ, TLCφ, and RS1φ, *Vibrio* pathogenicity islands 1 (VPI-1) and 2 (VPI-2), *Vibrio* seventh pandemic islands I (VSP-I) and II (VSP-II), as well as integrative conjugative elements of the SXT family, which carry the genes involved in multidrug resistance—showed high nucleotide sequence similarity to the same regions of the new hypervirulent El Tor variants isolated in 2010 from a cholera outbreak in Haiti (3, 5). In contrast to El Tor variants possessing the cholera toxin prophage CTXφ containing the classic allele of the *ctxB* gene, *ctxB1*, strain 76 carried a novel allele, *ctxB7*. The analysis of the VPI-1 region revealed that the *tcpA* gene gained an A_266_G mutation. Furthermore, a novel rearrangement of the VSP-II region with a 13.1-kb deletion was found in strain 76. The draft genome of strain M3265/80 shares >99.9% average nucleotide identity with the 76 genome. Phylogenetic analysis based on single-nucleotide polymorphisms located in core genes orthologous to genes in previously sequenced genomes deposited in GenBank showed that strains 76 and M3265/80 are directly related to outbreak isolates from India (2007), Bangladesh (2002 to 2007), and Haiti (2010). A more detailed analysis of these draft genomes will be the focus of a future publication. The whole-genome sequences of strains 76 and M3265/80 provide valuable insights into the evolution of cholera agents and will help improve disease control strategies.

### Accession number(s).

The whole-genome shotgun projects have been deposited in DDBJ/ENA/GenBank under the accession numbers MPVL00000000 (76) and JRQL00000000 (M3265/80). The versions presented in this article are the first versions.

## References

[1] Sack DA, Sack RB, Nair GB, Siddique AK 2004 Cholera. Lancet 363:223–233. doi:10.1016/S0140-6736(03)15328-7. 14738797

[2] Safa A, Nair GB, Kong RY 2010 Evolution of new variants of *Vibrio cholerae* O1. Trends Microbiol 18:46–54. doi:10.1016/j.tim.2009.10.003. 19942436

[3] Kim EJ, Lee CH, Nair GB, Kim DW 2015 Whole-genome sequence comparisons reveal the evolution of *Vibrio cholerae* O1. Trends Microbiol 23:479–489. doi:10.1016/j.tim.2015.03.010. 25913612

[4] Smirnova NI, Zadnova SP, Agafonov DA, Shashkova AV, Cheldyshova NB, Cherkasov AV 2013 Comparative molecular-genetic analysis of mobile elements in natural strains of cholera agent. Russ J Genet 49:898–908. doi:10.1134/S1022795413090081. 25486771

[5] Satchell KJ, Jones CJ, Wong J, Queen J, Agarwal S, Yildiz FH 2016 Phenotypic analysis reveals that the 2010 Haiti cholera epidemic is linked to a hypervirulent strain. Infect Immun 84:2473–2481. doi:10.1128/IAI.00189-16. 27297393 PMC4995894

